# Microarrays in ecological research: A case study of a cDNA microarray for plant-herbivore interactions

**DOI:** 10.1186/1472-6785-4-13

**Published:** 2004-09-07

**Authors:** Matthias Held, Klaus Gase, Ian T Baldwin

**Affiliations:** 1 Department of Molecular Ecology, Max-Planck-Institute for Chemical Ecology, Hans-Knöll-Str. 8, 07745 Jena, Germany; 2 Institute of Ecology, Friedrich-Schiller-University, Dornburger Str. 159, 07743 Jena, Germany

## Abstract

**Background:**

Microarray technology allows researchers to simultaneously monitor changes in the expression ratios (ERs) of hundreds of genes and has thereby revolutionized most of biology. Although this technique has the potential of elucidating early stages in an organism's phenotypic response to complex ecological interactions, to date, it has not been fully incorporated into ecological research. This is partially due to a lack of simple procedures of handling and analyzing the expression ratio (ER) data produced from microarrays.

**Results:**

We describe an analysis of the sources of variation in ERs from 73 hybridized cDNA microarrays, each with 234 herbivory-elicited genes from the model ecological expression system, *Nicotiana attenuata*, using procedures that are commonly used in ecologic research. Each gene is represented by two independently labeled PCR products and each product was arrayed in quadruplicate. We present a robust method of normalizing and analyzing ERs based on arbitrary thresholds and statistical criteria, and characterize a "norm of reaction" of ERs for 6 genes (4 of known function, 2 of unknown) with different ERs as determined across all analyzed arrays to provide a biologically-informed alternative to the use of arbitrary expression ratios in determining significance of expression. These gene-specific ERs and their variance (gene CV) were used to calculate array-based variances (array CV), which, in turn, were used to study the effects of array age, probe cDNA quantity and quality, and quality of spotted PCR products as estimates of technical variation. Cluster analysis and a Principal Component Analysis (PCA) were used to reveal associations among the transcriptional "imprints" of arrays hybridized with cDNA probes derived from mRNA from *N. attenuata *plants variously elicited and attacked by different herbivore species and from three congeners: *N. quadrivalis, N. longiflora *and *N. clevelandii*. Additionally, the PCA revealed the contribution of individual gene ERs to the associations among arrays.

**Conclusions:**

While the costs of 'boutique' array fabrication are rapidly declining, familiar methods for the analysis of the data they create are still missing. The case history illustrated here demonstrates the ease with which this powerful technology can be adapted to ecological research.

## Background

The 'genomics revolution' has provided the information needed to analyze how a genome responds to the environment in the formation of the "transcriptome", the portion of the genome that is transcribed. Microarrays, which offer the ability to analyze the expression ratios (ERs) of thousands of genes simultaneously, represent one of many new tools produced by this effort. However, not all biological disciplines have benefited equally from this technology, and array technology has not been widely adopted by the ecological community for a number of reasons. The large genome-wide arrays are only available for select model organisms, which may not be appropriate for many ecological questions. Moreover, the complexity of their analysis and the costs of the available commercial software solutions prohibit their adoption by small research groups and constrain the number of biological experiments that can be conducted even by large, better-funded groups. 'Boutique' arrays – on which a smaller fraction of the transcriptome, typically representing a selection (hundreds) of genes specific to a class of genetic elements or response types – are readily created for a non-model organism at costs that are affordable for small research groups. However, the problems remain of how best to normalize and analyze array data. A large number of software solutions are available but no clear best solution has emerged [1-5].

A recent review has examined the types of arrays as well as the ecological and evolutionary questions that can be addressed with microarrays [6]. Here we present a cDNA microarray designed to analyze plant-herbivore interactions in a native plant. A cDNA microarray is a comparative tool, providing relative ERs for multiple genes from two differentially labeled fluorescent cDNA samples prepared by reverse transcription of mRNA extracted from matched plant samples. Hence the procedure is particularly useful for the analysis of plant responses elicited by herbivore attack: the induced defense and tolerance responses of plants [7]. We examine a number of practical challenges facing the adoption of boutique arrays for ecological research with tools familiar to ecologists, including signal normalization, the use of arbitrary expression thresholds to determine the significance of expression, the use of within-and between-array signal variance in evaluating the effect of probe quality and quantity and array age, as well as data analysis and visualization by cluster and Principal Component Analysis (PCA).

The microarray was designed to examine herbivore-induced gene expression in the model ecological expression system, *Nicotiana attenuata *[8]. The genes for the microarrays were derived from a series of display (differential display reverse transcriptase-PCR, subtractive hybridization with magnetic beads, and cDNA-AFLP display) experiments that compared the transcriptome of plants attacked by the larvae of its specialist herbivore, *Manduca sexta*, with that of unattacked plants [9-11]. Two independent and differentially end-labeled cDNA probes of each of 240 genes were spotted in quadruplicate on each array. Hence each gene was represented by 8 replicate probes, which were used to analyze within-array ER variance (array CV). Since the array was composed of genes that were both down-or up-regulated in response to *M. sexta *attack, an array-specific normalization factor could be readily calculated. The effects of microarray age and cDNA quality on the measures of array CV were estimated. We present a 2-step criterion for determining significant expression based on t-tests of replicate ERs and arbitrary thresholds. We re-examine the use of arbitrary expression thresholds with a 'norm of reaction' analysis of 6 genes derived from the 73 hybridization experiments. The data from microarrays are frequently analyzed with cluster analysis procedures [12], which deliver a limited analysis of the statistical associations. PCA is frequently used in ecological studies but is not commonly used in the analysis of array data. We present a PCA of 35 hybridized arrays, which visualizes the contribution of ERs from particular genes to the associations among arrays in the PCA.

## Results and Discussion

### Array CVs, array age and probe quality

The array CV for each of the 73 arrays was strongly correlated with the number of gene ERs that showed higher values than the defined threshold for the variance (R^2 ^= 0.969, F_69,1 _= 2102, *P *< 0.001). This demonstrates that the array CV corresponds to the number of gene ERs that are outliers and therefore reflects the "quality" of the information derived from the array. We used array CVs to test if array age could explain some of the variance and found no detectable effect (R^2 ^= 0.025, F_69,1 _= 2.73, *P *= 0.103).

The spectrum of the cDNA was recorded between 240 and 700 nm. Shape and maxima of the curves for the particular compounds (DNA, Cy3, Cy5) allowed the evaluation of cDNA quantity and quality. The quantity of the cDNA that was hybridized was estimated by its OD at 260 nm. The quality of the fluorescently labeled probe derived from this cDNA was estimated by the relation of the quantity of the two dyes at 550 nm (Cy3) and 650 nm (Cy5) and the cDNA quantity. These linear regressions revealed that for cDNA quantity (OD 260 values), neither Cy3 nor Cy5 values were significantly correlated with array CVs (all R^2 ^< 0.007, all Ps > 0.225). There was a negative correlation between array CV and OD 550 values for Cy3 (R^2 ^= 0.069) and OD 650 values for Cy5 (R^2 ^= 0.051) with slopes of -7.55 and -5.7, respectively. However, only the Cy3 regression was significant (P = 0.028) whereas Cy5 was not (P = 0.06). A similar pattern was apparent for the probe quality: Cy3 and Cy5 quality parameters were negatively correlated with array CV, but only the regression for Cy3 (R^2 ^= 0.144, slope = -0.31) was significant (P = 0.001) whereas the regression for Cy5 (R^2 ^= 0.042, slope = -0.126) was not (P = 0.088). From this analysis, we conclude that the quality of the labeled cDNA sample to be hybridized to an array predicts the quality of the signals produced from the array.

### PCR product quality

**Figure 2:**
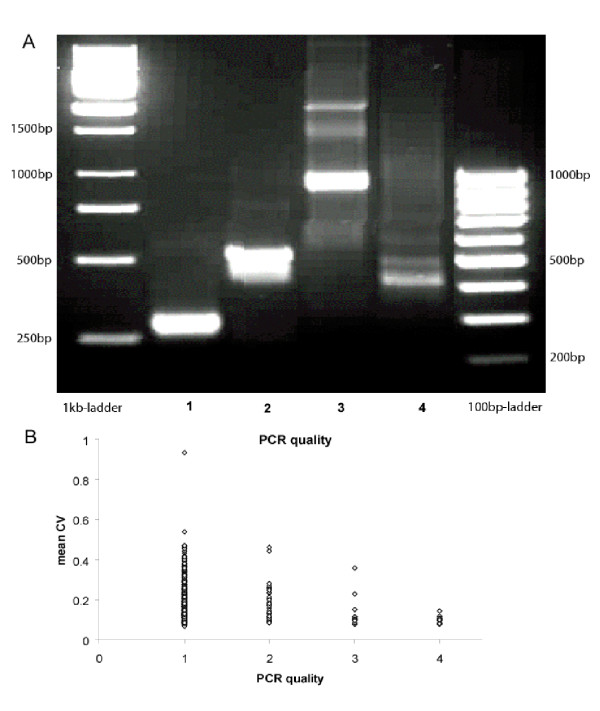
**A:** 2% agarose gel with 1 kb and 100 bp size ladders and examples illustrating the 4 different PCR-qualities classes (1 – 4): 1 = single band: 2 = single band with slight background indicating multiple non-specific PCR products; 3 = single band with strong background; 4 = multiple bands with background or only background. **B: **Mean coefficient of variance (CV) of expression ratios for 8 replicate cDNA products from 253 genes (array CV) measured from73 hybridized microarrays based on the 4 PCR quality classes described in A. Classes have significantly different CVs (Kruskal-Wallis ANOVA on Ranks, H_3 _= 40.603, *P *< 0.001)

The 502 different PCR products (2 for each gene + internal controls) that were spotted on the arrays had the following distribution in the 4 quality categories (Fig 2A): 1 = single band (426): 2 = single band with slight background (48); 3 = single band with strong background (14); 4 = multiple bands with background or only background (14). Multiple bands were only spotted to determine how low quality PCR products effect the results. To evaluate the association of PCR product quality on the variance of ERs, gene CVs were plotted against the PCR quality class. Gene CVs were found to be significantly different among the 4 PCR categories (Fig. 2B, Kruskal-Wallis ANOVA on Ranks, H_3 _= 40.603, *P *< 0.001). While *post hoc *tests revealed that PCR quality did not have a directional effect on gene CV, it was lowest for genes with intermediate CVs and increased in genes with high and low CVs. We conclude that the quality of the PCR product spotted on arrays does not have a strong effect on gene CV.

### Expression patterns

#### All arrays

**Figure 3:**
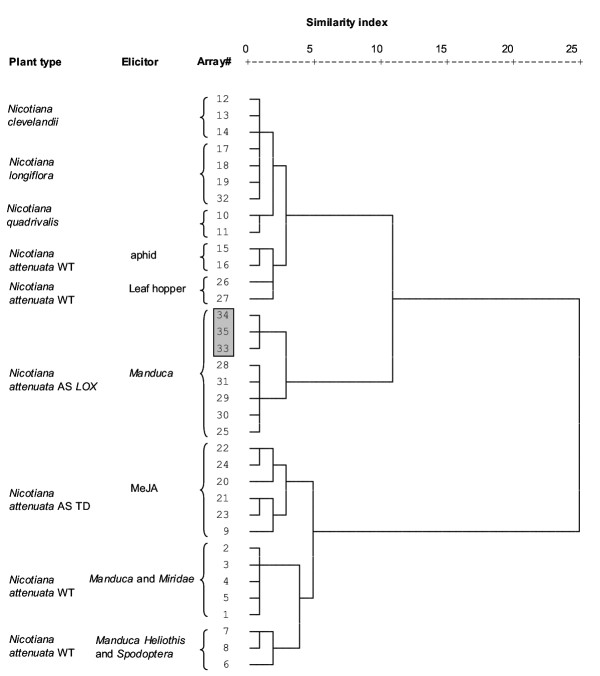
Cluster analysis (Ward's method, squared Euclidean distance) showing similarities between 35 hybridized microarrays hybridized with probes from wildtype (WT), antisense Lox-3 (AS *LOX*) and antisense TD (AS *TD*) of the diploid native tobacco species, *Nicotiana attenuata *plants and from untransformed plants of 3 congeneric *Nicotiana *species, two of which are allotetraploids *N. quadrivalis *and *N. clevelandii *and that are thought to have *N. attenuata *as a common ancestor as well as the more distantly related, *N. longiflora*. Arrays were hybridized with probes from plants attacked by different herbivore species or elicited with methyl jasmonate (MeJA). The shaded box represents 3 replicate hybridizations of the same sample of m-RNA from *LOX N. attenuata *plants. Arrays included in brackets correspond to clusters in the PCA (Fig. 4).

A cluster analysis of 35 arrays (Fig. 3) reflected the similarities of the transcriptional patterns observed in arrays hybridized with similar treatments. Arrays hybridized with probes derived from mRNA from *N. clevelandii *(arrays 12, 13, 14) and *N. longiflora *(arrays 17, 18, 19, 32) were separated from those hybridized with material from *N. quadrivalis *(arrays 10, 11) and *N. attenuata *that had been attacked by aphids or leaf hoppers (arrays 15, 16, 26, 27). These arrays were separated from those hybridized with samples from antisense-transformed *N. attenuata *plants that had been attacked by *Manduca *caterpillars (arrays 25, 28, 29, 30, 31, 34, 35), and the cluster they formed was separated from all other arrays that had been hybridized with *N. attenuata *material elicited by methyl jasmonate treatments (MeJA, arrays 9, 20, 21, 22, 23, 24) or attacked by *Manduca*, mirid, *Heliothis *or *Spodoptera *herbivores (arrays 1 – 8). The 3 replicated arrays hybridized with the same mRNA clustered together (arrays 33, 34, 35). The details of these similarities will not be treated here, as they are discussed in separate publications. The similarity of the elicited transcriptional signatures observed on the arrays hybridized with the *N. longiflora *and *N. attenuata *[13] probes demonstrates the utility of the array in the analysis of samples from congenerics.

**Figure 4:**
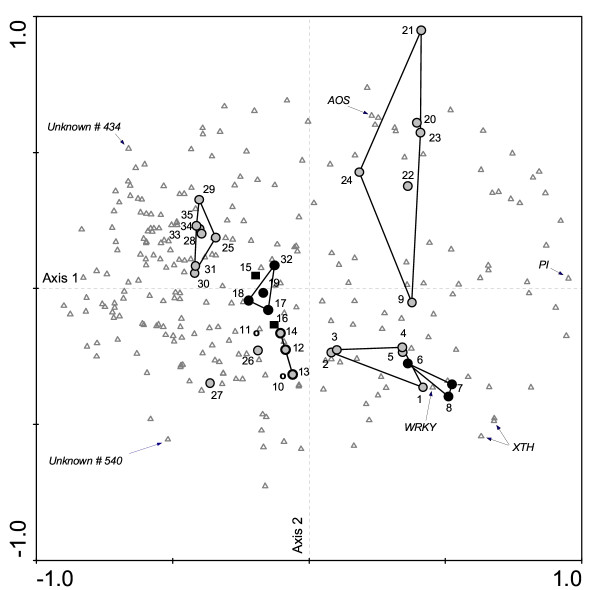
Principal component analysis (PCA) of the distribution of mean gene expression ratios of 234 genes (origin of vector is at the intersection of Axes 1 and 2 and the top of vector plotted as triangles) in the 35 hybridized microarrays (plotted as circles and squares) hybridized as described in Fig. 3. Arrays are labeled according to plant species and treatment (see Fig 3). Particular genes are identified with arrows. Clusters of microarrays with similar treatments are connected by lines. Axes 1 and 2 account for 40.1 and 8.3 % of the variation, respectively. Vectors of genes that are relatively long and parallel and, as such, correlated with the first canonical axis explain a large part of the variance. Vectors are strongly (up-or down) regulated in arrays and clusters of arrays lying near the end of a particular gene vector. For example, the *WRKY *gene vectors (originating at the intersection of Axes 1 and 2 and terminating at the *WRKY *gene triangle) contribute significantly to the cluster of microarrays hybridized with labeled cDNA derived from MeJA-elicited and *Manduca *and mirid attacked plants. Or, for example, the *AOS *gene vectors (originating at the intersection of Axes 1 and 2 and terminating at the *AOS *gene triangle) contribute significantly to the clusters of microarrays hybridized with labeled cDNA from MeJA-treated plant.

A PCA of the same 35 arrays (Fig. 4) showed a similar pattern of associations but provided the additional information of which genes contributed most to the patterns observed in the PCA. The vector of the gene coding for proteinase inhibitors (*PI*) was correlated with the first canonical axis that explained 40% of the total variance in the dataset. Moreover, transcripts for PI were up-regulated in the *N. attenuata *arrays elicited with MeJA or attacked by *Manduca*, mirid, *Heliothis or Spodoptera *herbivores. Expression of xyloglucan endo-transglycosylases (*XTH*) and *WRKY *transcription factor transcripts was also correlated with the first axis and correlated with the location of arrays 1 – 8 in the PCA. These 2 genes were plotted relatively close together in the PCA, reflecting their similar patterns of regulation across all arrays. The vector of allene oxide synthase (*AOS*) transcript expression reveals a correlation with arrays hybridized with mRNA from MeJA-elicited plants. *AOS *catalyzes a later stage in the biosynthesis of jasmonic acid and is known to be elicited by MeJA treatments [14]. The response of two unknown genes (*434 *and *540*) exemplifies genes whose pattern of expression is opposite to that discussed for the genes of known function. The ERs of *gene 434 *reacted in the opposite direction as those of *WRKY *and *XTH*, and the responses were larger in antisense *N. attenuata *plants. The response of *gene 540 *was opposite to that of *AOS *and larger in *N. clevelandii *and *N. attenuata *plants attacked by leaf hoppers.

#### Individual genes

**Figure 1:**
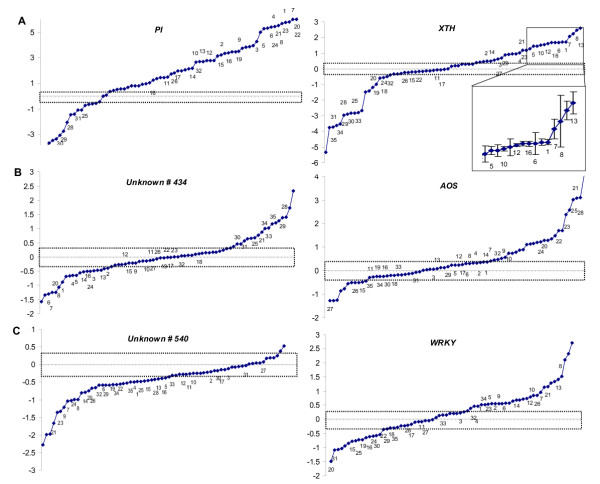
Norm of reaction of expression ratios (ER) for 6 genes from 73 hybridized microarrays of which the 35 presented in the cluster analysis (Fig. 3) are labeled with numbers. Distribution of log-transformed mean expression ratios of 4 genes of known function [proteinase inhibitor (*PI*), xyloglucan endo-transglycosylase (*XTH*), NtWRKY2 (*WRKY*) and allene oxide synthase (*AOS*)] and 2 of unknown function (*540 *and *434*); dotted areas represent the arbitrary ER thresholds of 1.24 and 0.81 (corresponding to ± 0.3 for log_2 _transformed values). Genes are organized according to the relative spread of their expression ratios A > B > C. Insert in *XTH *panel shows error structure (mean ± SE) on a non-log scale calculated from 8 replicate spots from each array.

The expression patterns of 6 genes (4 of known function; 2 of unknown function), as the mean of 2 PCR fragments with differently modified primers across 73 experiments, illustrate the 'norm of reactions' of the transcriptional responses of these genes (Fig. 1). The transcriptional responses of these genes were in opposite directions and within various ranges of expression to the different treatments. Genes such as *PI *and *XTH *exhibit strong up-regulation (up to 88-fold) in response to herbivore attack and jasmonate elicitation, and are similarly strongly down-regulated (50-fold) when plants transformed to silence endogenous jasmonate biosynthetic enzymes (antisense*LOX *[15]) are elicited and compared with untransformed plants on the same array. The inset of the *XTH *norm of reaction depicts the variance in ERs from a selection of individual arrays to illustrate that treatments (arrays 16 and 6) eliciting very similar mean ERs (both 3.20) can have very different gene standard errors of the mean ER (SE) (0.19 and 0.78 respectively). Shaded areas represent the arbitrary ER thresholds of ± 0.3 for log_2_-transformed values. All 6 genes had numerous treatments in which these thresholds were exceeded, but the genes differed in the magnitude and direction in which the threshold ERs were exceeded. In contrast to the *PI *and *XTH *genes, the unknown *gene 540 *and the *WRKY *transcription factor had more attenuated 'norm of reactions', being maximally up-and down-regulated by only 6.5-and 5-fold across all 73 arrays. In a majority of the experiments, the *AOS *gene was up-regulated, while down-regulation was more common for unknown *gene 540*. In many experiments, however, ERs did not exceed threshold values.

Ecologists are frequently interested in the processes that "fit" organisms to their environment. Adaptation to a particular environment results in part from the phenotypic consequences of hundreds of coordinated changes in gene expression, but because many levels of organization exist between an organism's transcriptome and its phenotype, it is often unclear how best to study the process of adaptation. Array technology has the potential to identify genes relevant to the process of adaptation, regardless of the time scale involved (evolutionary to physiological). However, a number of technical issues remain to be solved before the technology can be fully incorporated into ecological research: the normalization of signals, the within-and between-array variability of ERs, and the general problem of coping with the large amount of data that array studies produce. Many techniques have been discussed but a consensus for a standard solution [4] has not yet emerged.

#### Normalization

Since mRNA samples are labeled with different efficiencies and the different fluorescent dyes have different optical properties, signals from an array require normalization before ERs can be calculated. The literature addressing the problems of normalization has been reviewed [1,2], with the consensus conclusion that there is no single best way to normalize array data and that specific solutions were required for the particularities of each array. When arrays are created with cDNAs that are typically both up-and down-regulated, a total intensity normalization can be used. By adapting a total intensity procedure [1] we normalized the signals from only the middle 75% of the distribution from a given array which produced values that were highly comparable among arrays, as demonstrated by the similarity of the clustering of the 3 replicate arrays (arrays 33, 34, 35; Fig 3, Fig. 4).

#### Variance

ERs from microarrays are derived from two differently labeled but mixed samples that competitively hybridize to immobilized gene-specific probes. The outcome of this hybridization can vary substantially within an array, as measured by the variance in ERs measured across replicate spots. The strong positive correlation between the number of genes above the specified ER-threshold and the array CVs highlights the utility of array CVs to summarize the quality of a given hybridization. Little is known about the factors that influence within-array hybridization or the amount of spot replication that is required to cope with the variance typically found in environmental samples [16]. However, the 8 replicate spots for each gene distributed across the array provided valuable data on gene and array CVs. From these CVs we were able to determine the quality of ER patterns from single arrays and single genes.

Most of the technical parameters tested were not correlated with the variance structure in our dataset. Our measures of PCR product quality did not explain the variance of gene CVs. Similarly, array age did not account for a significant amount of variation in array CVs. In contrast, probe quality was negatively correlated with array CV and explains a part (ANOVA, F_69,1 _= 5.046, *P *= 0.028) of the variance in array CVs. In our data set, a 15-fold increase in OD was associated with a halving of array CV. Therefore the monitoring of this measure of probe quality could save the costly use of arrays for samples that will likely produce low-quality results. Since none of the measured parameters unambiguously explained the pattern of within-array variance in our dataset, we conclude that a combination of several factors including the probe quality determines array variance.

#### Data analysis

Cluster analysis revealed groups of treatment that resulted in similar patterns of expression and, in doing so, provided a visual demonstration that the results obtained were reproducible. The PCA proved to be more useful for exploratory data analysis than did cluster analysis, because it provided information on the single gene vectors that contributed to similarities and differences among arrays. PCA allows researchers to quickly visualize similarities in expression patterns between known and unknown genes, and thereby generates hypotheses about the function and regulation of genes of unknown function. For example, in our analysis, a group of unknown genes – from which we chose two (*434 and 540*) as proxies – explained a relatively large part of the variance (indicated by long vectors) and was positively correlated to specific treatments and negatively correlated to vectors of genes of known function. *Gene 434 *was up-regulated in antisense *LOX *plants and had the opposite pattern of expression compared to that of the *WRKY *and *XTH *genes, both of which are strongly up-regulated by herbivore attack and jasmonate elicitation. *Gene 540 *had the opposite pattern of regulation as did *AOS *with higher ERs in plants attacked by leaf hoppers, suggesting a role in the plants' response to this herbivore. The PCA of Fig. 4 is a 2-dimensional presentation of a multidimensional analysis and analyses that allow for multidimensional presentations of the associations, provide more accurate information on the contribution of single gene vectors to associations among arrays.

Quantitative geneticists have coined the term 'norm of reaction' for the variation in phenotypic expression of a given genotype across a number of different environments. We apply this term to characterize the range of ERs observed for a given gene across a number of different expression experiments. The information provided in a norm of reaction provides a biologically informed alternative to the use of arbitrary thresholds for the determination of significant expression. This would allow researchers to use lower thresholds for genes (e.g. *WRKY *transcriptions factors) that are known to show low dynamic ranges of expression and higher thresholds for genes with likely larger dynamic ranges, such as those directly involved in defense metabolite production (e.g. PI). Additionally, when comparing many arrays, a norm of reactions provides information that allows researchers to determine if a given array is providing ERs within the normal range of variance found in prior experiments.

## Conclusions

We conclude that the data produced by 'boutique' microarrays can be readily analyzed with inexpensive home-grown procedures that are commonly used in ecological studies. Arrays with sufficient within-array replication allow for the calculation of gene and array CVs that are useful in estimating the quality of the information gathered from a given array. Furthermore, multivariate statistical techniques, such as PCA, can be used to visualize global expression patterns and identify the individual genes that make large contributions to the transcriptional signatures of particular treatments. The costs of boutique arrays are approaching those of many standard ecological procedures, and the information they provide will allow ecological researchers the ability to characterize early stages in an organisms' response to environmental changes.

## Methods

### Microarray construction and hybridization

The cDNA microarray and its hybridization is described in [11], and a complete list of cDNAs and their physical location on the microarray can be found at: [11] (supplemental Table I at ). Briefly, the production of the cDNA microarray started with a set of 234 genes which were cloned by differential display reverse transcription (DDRT)-PCR and subtractive hybridization using magnetic beads (SHMB) of *M. sexta *larvae-attacked *N. attenuata *plants [9,10] or by cDNA-AFLP (amplified fragment-length polymorphism) display of *N. attenuata *plants under simulated *M. sexta *attack by applying oral secretions and regurgitant to leaf wounds [11] and 6 well-characterized *Manduca*-induced genes (putrescine methyl transferase, xyloglucan-8 endotransglycosylase, allene-oxide synthase, hydroperoxide lyase, trypsin inhibitor, *WRKY *transcription factor). These genes were PCR amplified and for each cDNA, two PCR fragments, with 5'-aminolink on either strand, were synthesized. Each PCR fragment was robotically spotted four times on epoxy coated slides (Quantifoil Micro Tools GmbH, Jena); hence, each gene was represented on the microarray 8 times: by two independent PCR fragments, which, in turn, were each spotted in quadruplicate.

The cDNA microarrays were hybridized with fluorescently labeled cDNA prepared by reverse transcription of mRNA isolated from leaf tissues of 73 differently elicited *Nicotiana *plants belonging to 4 species. Competitive hybridization of 2 samples (treated and untreated plants) with different dyes (Cy3 and Cy5) defined the ratio of transcript abundance in the treatment sample compared to the control sample for each spot on the microarray. A majority of the arrays were hybridized with samples from wildtype or transformed [17]*N. attenuata *plants, which were elicited by attack from either various herbivore species (larvae of *Manduca*, *Heliothis*, *Spodoptera *moths, and adults and nymphs of aphids and mirids that attack *N. attenuata*), methyl jasmonate (MeJA), or larval regurgitant treatments or UV-B exposure, and compared with plants of the same genotype, age, and developmental stage which were unelicited. To determine the utility of the arrays in the analysis of responses from congenerics, arrays were hybridized with samples taken from two tetraploid species (*N. quadrivalis *and *N. clevelandii*) that had evolved from independent allopolyploid hybridizations between *N. attenuata *and another extinct 12-chromosome *Nicotiana *taxa [18], as well as the more distantly related, *N. longiflora*. The details of each hybridization and the specific gene responses of the arrays are either published [11,13,19] or are in preparation. Here we present a global analysis of 73 arrays to identify methods of analysis for such boutique microarrays that are useful for ecological research.

### Normalization and statistics

Because the arrays included both up- and down-regulated genes, the calculation of a microarray-specific normalization factor provided a valuable alternative to the use of external reference controls, which may or may not be influenced by the elicitation conditions [2,20-22]. The measured Cy5 and Cy3 fluorescence intensities were ranked independently, and after discarding the 12.5% maximum and minimum values, the remaining 75% of the values were summed (adapted total intensity normalization, [1]). The array-specific normalization factor was obtained by dividing the calculated sum of Cy3 values by those of the Cy5 values. The ratios of normalized fluorescence values for Cy3 and Cy5 of each individual spot (expression ratio = ER) and the mean of the four replicate spots for each cDNA (2 for each gene = ER1, ER2) were calculated. ERs were subjected to a t-test to determine if the values differed significantly from 1. A transcript was defined as being differentially regulated if both of the following criteria were fulfilled: 1) the final ER (ER1+ER2)/2) was equal to or exceeded the arbitrary thresholds [≤ 0.81 (log_2_0.81 = -0.3) for down-regulated genes or ≥ 1.24 (log_2_1.24 = 0.3) for up-regulated genes]; 2) both ER1 and ER2 were significantly different from 1 as evaluated by t-tests to control for ER-variance and ER-sample size. An arbitrary threshold was utilized for two reasons: first, to account for normalization errors, and second, to account for the fact that replicate data did not result from repeated hybridizations with the same RNAs but from repeated probe spotting.

To evaluate our criteria, we hybridized three arrays with the same cDNA pools and found that 210 of 234 genes (84%) had the same regulation identified by the criteria described above. Of the 30 genes that did not show consistent regulation between the two repeated hybridizations, 24 had the same direction in mean ER but did not meet the statistical requirements for a significant change. These 3 replicate arrays were located together in both the cluster analysis (Fig. 3) as well as the PCA (Fig. 4). To further estimate the variance of ERs, the mean coefficient of variation (CV) was calculated for each of the genes (gene CV) and each of the arrays (array CV). Gene CVs were obtained by calculating the mean of the individual CVs for each gene on each array; they were used to evaluate the effects of PCR product quality and the thresholds were used to determine significant expression. The quality for each gene was regarded as too low when its mean CV (mean of all 73 arrays) was higher than 0.3. Gene CVs are not influenced by the absolute expression values and reflect the variation across replicate spots on a given array. Array CVs were calculated as the mean of all individual gene CVs for each array and were used to evaluate array and cDNA quantity and quality that was hybridized to the arrays. Since the 73 arrays analyzed in this study were hybridized over 8 months after the arrays were spotted, we used array CV to assess array ageing.

A cluster analysis of 35 arrays was performed based on Ward's method and the squared euclidian distance [23,24]. To evaluate the appropriate model for the description of the gene distribution, a Detrended Correspondence Analysis (DCA) was performed. The given dimensionless value for the length of gradient of the first ordination axis was < 1.8, which indicated that the values were better fitted by a linear (lg < 3) than a unimodal (lg > 4) distribution model [25]. Therefore, a PCA based on a linear model was chosen to compare gene expression within the microarrays. PCA was performed on log-transformed mean expression ratios of all transcripts from a sample of 35 arrays. Scaling was focused on inter-array distances. Four genes of known function and two of unknown function – from the PCA analysis (Fig. 4), these proved to be good discriminators of the arrays – were selected to calculate a gene-specific 'norm of reaction'. For this analysis, mean expression ratios for both PCR-fragments of each of these 6 genes over all arrays were calculated and hierarchically ordered on a log-based scale. For one of these genes (*XTH*), the error structure on a non-log scale is presented (Fig. 1 and inset).

To test for differences between the groups of different PCR qualities, Kruskal-Wallis ANOVA on Ranks was used. Test statistics and cluster analyses were performed with SPSS 11.0, PCA was carried out with the Canoco 4.5 package [25].

## Author's contributions

MH carried out the analysis of the microarray data, KG supervised the molecular work. ITB conceived of and coordinated the project, and ITB and MH wrote the manuscript.
